# Comparison of 1480 nm and 980 nm-pumped Gallium-Erbium fiber amplifier

**DOI:** 10.12688/f1000research.50952.2

**Published:** 2021-09-08

**Authors:** Siti Azlida Ibrahim, Amilia Mansoor, Tuan Ainin Sofea Tuan Mohd Marzuki, Nasr Y. M. Omar, Hairul Azhar Abdul Rashid

**Affiliations:** 1 Faculty of Engineering, Multimedia University, Cyberjaya, Selangor, 63100, Malaysia

**Keywords:** optical fiber, fiber amplifier, Erbium, Gallium

## Abstract

**Background:** One way to reduce the length of the gain medium in Erbium-Doped Fiber Amplifier (EDFA) is by doping the fiber core with a high concentration of Erbium. However, this method caused ion clustering effects, which limits the EDFA’s efficiency. In this research, the use of Gallium as a new co-dopant in erbium-doped silica fiber is explored.

**Methods:** The new fiber, namely Gallium co-doped Erbium fiber (Ga-EDF), is used as a gain medium in an optical fiber amplifier setup. A 2-meter length of the Ga-EDF fiber was used in a single pass configuration with a forward pumping scheme at 150 mW pump power. The Ga-EDF amplifier's gain and noise figure while pumping at 980 nm and 1480 nm were compared. The amplifier's performance was evaluated as the input signal power varied between -30 dBm to 3 dBm, over the wavelength range of 1520 nm to 1580 nm.

**Results:** The 980 nm-pumped Ga-EDF amplifier achieved the maximum small-signal gain of 22.45 dB and the corresponding noise figure of 5.71 dB at the input signal wavelength of 1535 nm. Meanwhile, the 1480 nm-pumped Ga-EDF amplifier attained the maximum small-signal gain of 20.83 dB and the corresponding noise figure of 5.09 dB at the input signal wavelength of 1550 nm. At the input signal power below -20 dBm and the wavelength range 1520 nm to 1547 nm, the Ga-EDF performs better when pumped at 980 nm. Their performance is comparable at the input signal wavelength range between 1547 nm to 1580 nm. At the input signal power above -20 dBm, the 1480 nm-pumped Ga-EDF outperformed the 980 nm-pumped amplifier.

**Conclusions:** The overall performance indicates that the gain saturation point of the 1480 nm-pumped amplifier is higher than the 980 nm-pumped.

## Introduction

Since its invention in 1987, Erbium-doped fiber amplifiers (EDFAs) have become the most crucial optical communication device in enabling the high-speed long-haul optical fiber communications system. The essential characteristics of EDFAs include the high optical gain in the C-band and L-band, high saturation output power, low noise figure, wide gain bandwidth, independent of bit rate, and insensitivity to polarization effects. Research in designing a compact, highly efficient EDFA is still ongoing. The optical fiber core is highly doped with Erbium ions to achieve high gain using a short gain medium. However, a high concentration of Erbium causes the clustering effect, which causes pair induced quenching and degrades the EDFA's performance. The clustering problem can be reduced by increasing the Erbium solubility in silica during the fiber fabrication process. The Erbium solubility can be improved by introducing a co-dopant such as Aluminium (Al) in the silica fiber ( Myslinski *et al.*, 1997). Since Gallium belongs to the same group of elements in the periodic table as Al, it is expected that its chemical properties will be somewhat similar to Al. The properties of Gallium oxide (Ga ^2^O _3_) as a glass-forming oxide and potential index riser make it suitable as a co-dopant.

Gallium co-doped Erbium fiber (Ga-EDF) was fabricated using modified chemical vapor deposition (MCVD) with a solution doping method to study its properties and potentials as a gain medium of a compact optical fiber amplifier ( Dissanayake *et al.*, 2015). The Erbium concentration and the Ga:Er ratio were approximately 2008 ppm and 17, respectively. The numerical aperture (NA) and the cut-off wavelength of the fiber were calculated to be 0.12 and 1400 nm, respectively. The core and cladding diameters were 9 µm and 125 µm, making it easy to splice to a standard single mode fiber. Recently, Ga-EDF's use as the gain medium in 860 femtoseconds mode-lock fiber laser has improved its performance ( Zazali *et al.*, 2019).

For EDFA with a high Erbium concentration, a 980 nm pumping scheme is more efficient than a 1480 nm pumping scheme ( Kimura & Nakazawa, 1993). However, a recent study showed that an amplifier made of a gain medium that is highly doped with Erbium and co-dopants, namely Hafnium and Bismuth, achieved higher gain using a 1480 nm pump ( Almukhtar *et al.*, 2019). It shows that the co-dopants play a crucial role in determining the fiber amplifier's most suitable pump wavelength. Recently, we reported on an amplifier with a single-pass configuration using a 2 m length of the Ga-EDF as a gain medium with a 980 nm forward pumping scheme ( Marzuki *et al.*, 2020).

This research aims to characterize and compare the performance of the Ga-EDF amplifier pumped at two different wavelengths, which are 980 nm and 1480 nm. This paper aims to discuss pump wavelengths' influence on the Ga-EDF amplifier's gain and noise figure at small-signal input and large-signal input.

### Literature review

**Figure 1.**
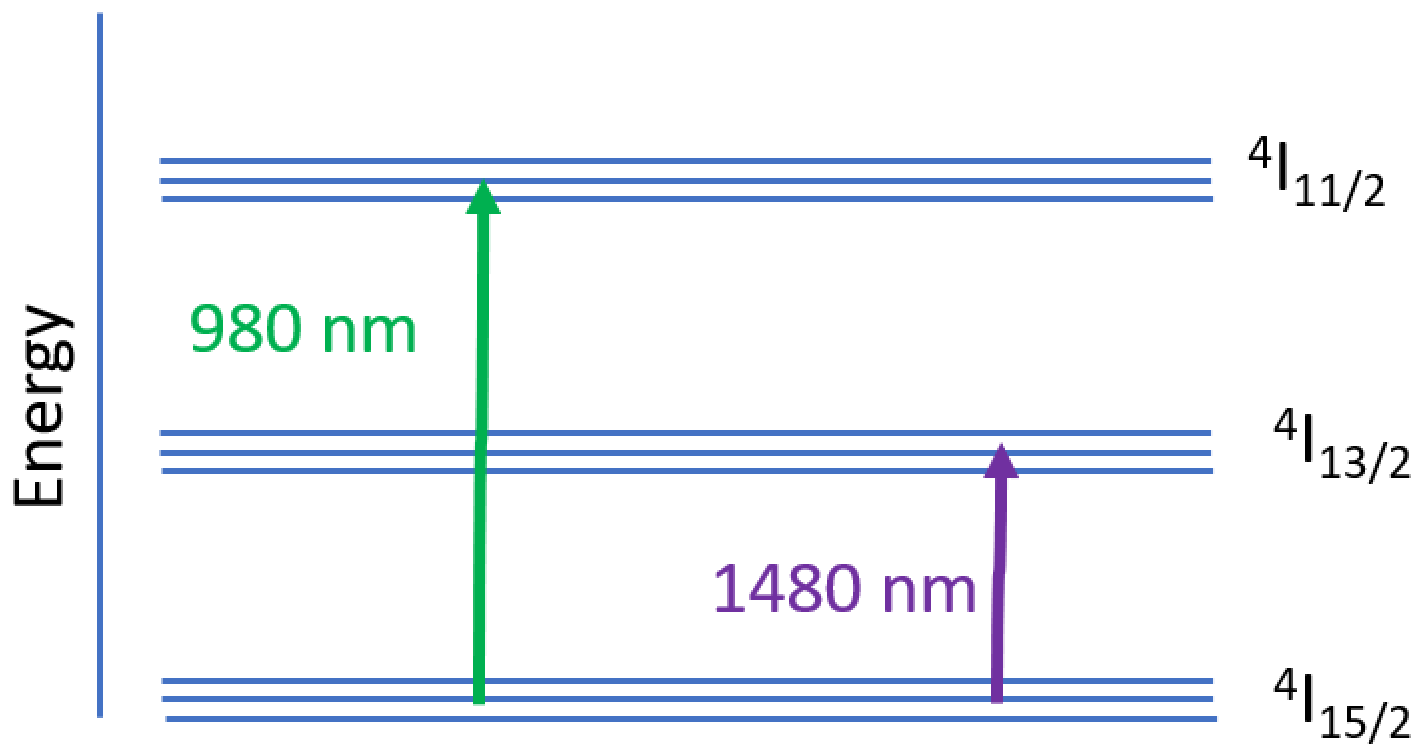
Three-level energy state model of Erbium ion.

The optical signal amplification process at different wavelengths in EDFA is best described using the three-level energy level model, as shown in Figure 1.

 In an optical fiber amplifier, pumping is a process to excite Er ^3+^ ions from the ground state to the upper energy level to cause population inversion. Pumping at 980 nm in EDFA is also known as indirect pumping, will excite the ions from the ground state ^4^I _15/2 _to the upper energy level ^4^I _11/2_. Since the upper energy level is not stable, the ions will rapidly decay to the metastable state energy level ^4^I _13/2_. This process is nonradiative, in which the energy is dissipated as heat. The ions' lifetime at the upper-state ^4^I _11/2 _in EDFA is very short, approximately 1µs. The ions lifetime at the metastable state ^4^I _13/2 _is much longer than the upper-state, typically around 10 ms. As a result, the continuous pumping process causes the accumulation of ions at the metastable state, and finally, the population inversion is attained. When the photons of the input signal enter the fiber, it causes the ions at the metastable state ^4^I _13/2_ to decay to the ground state ^4^I _15/2 _while releasing energy in the form of photons of the same phase and wavelengths. This radiative relaxation process amplifies the input signal, also known as the stimulated emission process. Erbium ions E+3's energy bands enable the amplification of signals in the C band wavelength range ( Desurvire, 2002).

Pumping at 1480 nm excites the ions from the ground state ^4^I _15/2 _directly to the metastable state ^4^I _13/2_. It provides a better power conversion efficiency in silica-based EDFA than the 980 nm pump ( Naji *et al.*, 2011). However, higher pump power is needed using 1480 nm compared to a 980 nm pump to achieve the same small-signal gain level. The noise figure of EDFA pumped at 1480 nm is also higher than the 980 nm for a specific small-signal gain ( Pedersen *et al.*, 1992). The absorption band of ^4^I _15/2 → _^4^I _13/2 _is quite broad. So, the pump laser wavelength does not need to be carefully selected. However, the overlapping of absorption and emission band causes incomplete population inversion, which causes unavoidable noise ( Miniscalco, 1991). The 1480 nm pump signal can travel over a longer distance in the silica optical fiber without a significant loss compared to the 980 nm signal. Therefore, a 1480 nm pump is preferred for remotely pumped amplifiers, such as in submarine applications.

 The amplified spontaneous emission (ASE) characteristic of a fiber amplifier is an essential factor that needs to be considered in designing an amplifier. Spontaneous emission happens when the ions at the excited state decay to the ground state and emit photons spontaneously. The spontaneously emitted photons then generate more photons as they propagate in the fiber. These photons add to the input signal as background noise, known as ASE noise. ASE behavior depends on the pump wavelength. The same fiber pumped at different wavelengths will show different ASE characteristics. The ASE also depends on the dopant composition. Pumping a YttErbium-Erbium doped fiber at 940 nm has been observed to suppressed the ASE ( Booker *et al.*, 2018). In this work, the ASE characteristics of Ga-EDF, forward pumping at 980 nm and 1480 nm, will be compared. Furthermore, ASE's influence on the Ga-EDF amplifier's gain and noise figure will also be analyzed. 

One of the critical performance characteristics for an optical fiber amplifier is the power conversion efficiency, which is the ratio between the output signal power over the input pump power. A few dissipative processes reduce the power conversion efficiency of the EDFA, namely multiphonon emission, excited-stateaAbsorption (ESA), and cooperative up-conversion. Multiphonon emission results in nonradiative relaxation, where no photons are generated. ESA is also a dissipative process where the ion is excited from the metastable state to a higher state through absorption of a pump or signal photon. Another dissipative process is the ion-ion interaction known as cooperative up-conversion ( Miniscalco, 1991). The cooperative up-conversion happens when two Erbium ions at the metastable state ^4^I _13/2 _exchange energy, which cause one ion to be excited to upper state ^4^I _19/2_, and another ion would decay to the ground state ^4^I _15/2_, without photon emission ( An *et al.*, 1998). This process causes concentration quenching, limiting Erbium's concentration in the EDFA. Erbium's low solubility in silica, which causes clustering, is the cooperative up-conversion's primary source. The suggested concentration for Erbium in silica is below 100 ppm for optimum amplifier efficiency ( Digonnet, 2001). Hence, a long gain medium is needed to achieve a specific gain, creating a bulky footprint EDFA. 

In designing a compact fiber amplifier using a short gain medium, many efforts were made to minimize the concentration quenching effect. One of the effective methods to increase Erbium solubility is by adding Aluminum to the silica host. Using 8000 ppm Aluminum and 8900 ppm Erbium, a 50 cm long EDFA has been found to achieve 24 dB gain with a pump power of 60 mW ( Kimura & Nakazawa, 1993). The ion clustering effect can also be reduced by adding other rare earth co-dopants such as Ytterbium and Lanthanum ( Baker *et al.*, 2019). A small-signal gain of 36.6 dB has been achieved using 1 m length Hafnium Bismuth EDF with 12500 wt ppm Erbium in the double-pass configuration ( Almukhtar *et al.*, 2019). A 1 m amplifier made of zirconia-yttria-alumina-baria silica host achieved a flat small-signal gain of 25 dB ( Duarte *et al.*, 2019). In chalcogenide glass fiber, Gallium has been used to prevent clusterings ( Digonnet, 2001). The solubility of rare-earth ions in the chalcogenide glass was increased with the presence of Gallium. Researchers at our research center conducted the first attempt to use Gallium as a co-dopant in a silica host by fabricating the Ga-EDF fiber using the MCVD and solution doping method ( Dissanayake *et al.*, 2015). The absorbance, fluorescence lifetime, and ASE were characterized ( Dissanayake, 2015). However, no analysis was done on the amplifier's performance at different pump wavelengths.

**Figure 2.**
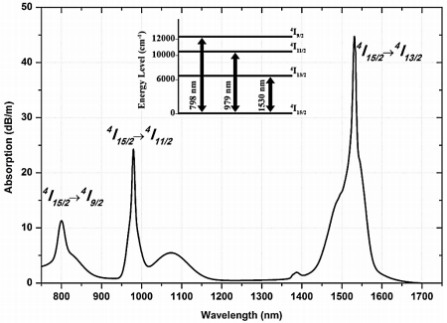
The absorption spectrum of Gallium co-doped Erbium fiber (Ga-EDF). This figure has been reproduced with the permission from Dissanayake (2015).

We continued the research to study the Ga-EDF detail characteristics. We recently demonstrated an amplifier with a small-signal gain of 22.45 dB using a 2 m Ga-EDF in a single-pass configuration ( Marzuki *et al.*, 2020). Figure 2 shows the absorption spectra for the Ga-EDF reported by Dissanayake *et al.* (2015). The spectra indicate that the Ga-EDF absorption is high at the standard pump wavelengths, 980 nm and 1480 nm. In this paper, the two pump wavelengths' influence on this amplifier's performance will be presented.

## Methods

**Figure 3.**
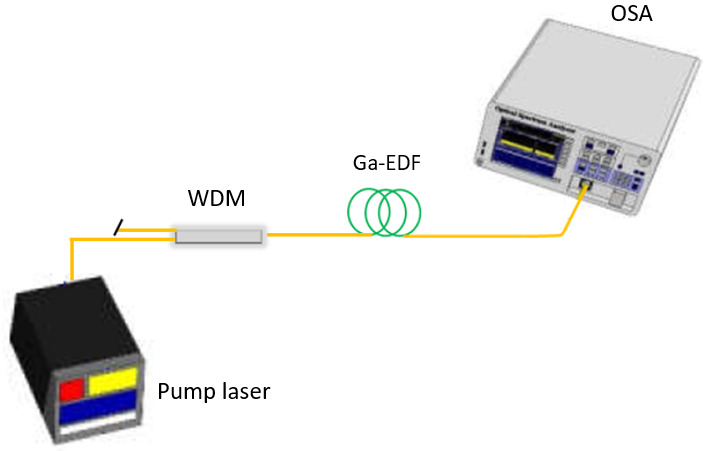
Experimental setup to characterize the amplified spontaneous emission (ASE) spectra of the Gallium co-doped Erbium fiber (Ga-EDF). WDM=wavelength division multiplexing coupler module; OSA=optical spectrum analyser.

Before the amplifier's gain and noise figure characterizations, the Ga-EDF's ASE spectra pumped at both pump wavelengths was measured. The experimental setup for the characterization of ASE spectra is illustrated in Figure 3.

A Lumics 980 nm 14-pin Butterfly LD powered by a Newport current and temperature controller (model 8000; Newport, Irvine, CA), together with a Lightel 980 /1550 nm wavelength division multiplexing (WDM) coupler module, was connected to the input end of the Er/Ga fiber. The Ga-EDF fiber's output end was connected to a Yokogawa ANDO AQ6370C Optical Spectrum Analyser (OSA) to measure the ASE spectra. The forward pump power was set to 150 mW before the measurement, and the OSA resolution was set to 0.2 nm. The experiment was repeated using Keopsys Continuous Erbium Broadband Source and a 1480/1550 nm WDM module to pump the Ga-EDF fiber at 1480 nm.

**Figure 4.**
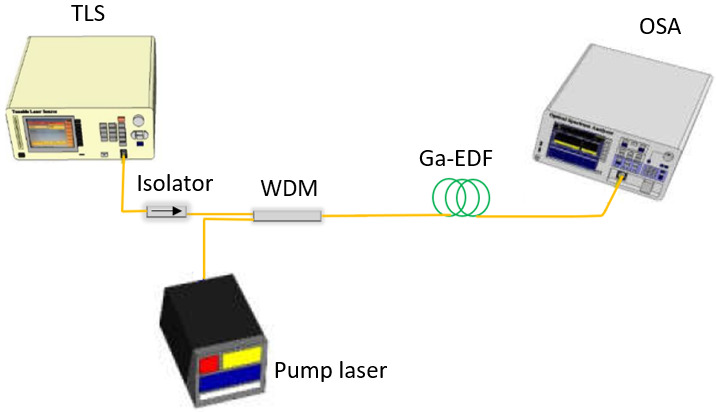
Experimental setup of the Gallium co-doped Erbium fiber (Ga-EDF) amplifier using a single-pass configuration with a forward-pumping scheme. TLS= Tunable Laser Source, WDM=wavelength division multiplexing coupler module, OSA=optical spectrum analyser.

Figure 4 shows the single-pass forward pumping optical fiber amplifier's experimental setup, with the 2 m length of Ga-EDF as the gain medium. The Ga-EDF was fabricated in-house using MCVD with the solution doping method, as described in Dissanayake (2015). The fiber core and cladding diameters are 9 µm and 125 µm, respectively. The cut-off wavelength for single-mode operation is 1.4 µm. ANDO 4321 C-band Tunable Laser Source (TLS) generates the input signal in the wavelength range of 1520 nm to 1580 nm, with the input signal power varied between -30 dBm to 3 dBm. An isolator was inserted between the TLS and WDM coupler to prevent the back-reflected signal from damaging the TLS. Lumics 14-pin Butterfly Laser Diode (LD) (Lumics, Berlin, Germany) was used as the pump laser at 980 nm. Keopsys 1480 nm Pump Source was used for 1480 nm pumping. The Newport current and temperature controller model 8000 controlled the 14-pin butterfly 980nm LD pumping the Ga-EDF at a maximum power of 150 mW. A 980/1550 nm WDM module was used to couple both input and pump signals into the Ga-EDF. The Yokogawa AQ6370D OSA with a resolution of 0.05 nm was used to measure the amplified signal across the wavelength range of 1500 nm to 1600 nm. The gain and noise figure was analyzed for the small-signal input power (-30 dBm) and large-signal input power (3 dBm).

The summary of the experimental parameters used in this research is listed in Table 1.

**Table 1.** Summary of experimental parameters. Ga-EDF=Gallium co-doped Erbium fiber, OSA=optical spectrum analyser, ASE=amplified spontaneous emission.

*Parameter*	*Value*
Pump power	150 mW
Ga-EDF length	2 m
Input signal power	-30 dBm to 3 dBm
Input signal wavelength	1520 nm to 1580 nm
OSA resolution for ASE measurement	0.2 nm
OSA resolution for gain and noise figure measurement	0.05 nm

## Results and discussion

**Figure 5.**
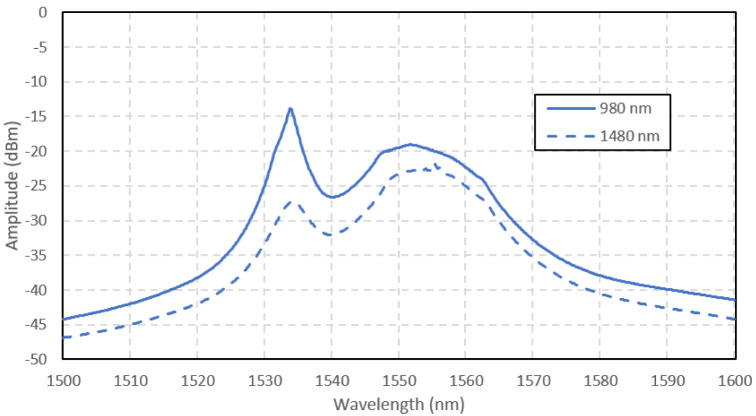
Amplified spontaneous emission (ASE) spectra of Gallium co-doped Erbium fiber (Ga-EDF) pumped at 980 nm and 1480 nm.

The ASE spectra of the Ga-EDF amplifier are shown in Figure 5 ( Ibrahim *et al.*, 2021). The amplitude of ASE was higher at 980 nm pumping because of a higher absorption at 980 nm than 1480 nm in the Ga-EDF, as characterized by Dissanayake *et al.* (2015). The absorption was 25 dB/m at 980 nm and 14 dB/m at 1480 nm. Higher absorption causes a higher population inversion to occur with the 980 nm pumping. From the measurement done by Dissanayake *et al.* (2015), the Ga-EDF fiber's fluorescence lifetime was 6.02 ms and 6.06 ms for 980 nm pump and 1480 nm pump, respectively. The shorter lifetime also contributes to the higher ASE at 980 nm pumping. As a result, the 980 nm-pumped amplifier's ASE amplitude was higher for the whole range of the measured signal wavelengths. The 980 nm-pumped amplifier has a peak ASE power of -16 dBm at 1535 nm, 11 dB higher than the 1480 nm-pumped signal. The two peaks (at 1535 nm and 1553 nm) represent the photons emission caused by the ions transition between the sub energy levels of the ^4^I _13/2 _metastable state and the ^4^I _15/2 _ground state. The peak wavelength of 1535 nm might be the Gallium co-dopant attribute in this fiber, which differs from the peak wavelength for EDF with other co-dopants.

**Figure 6.**
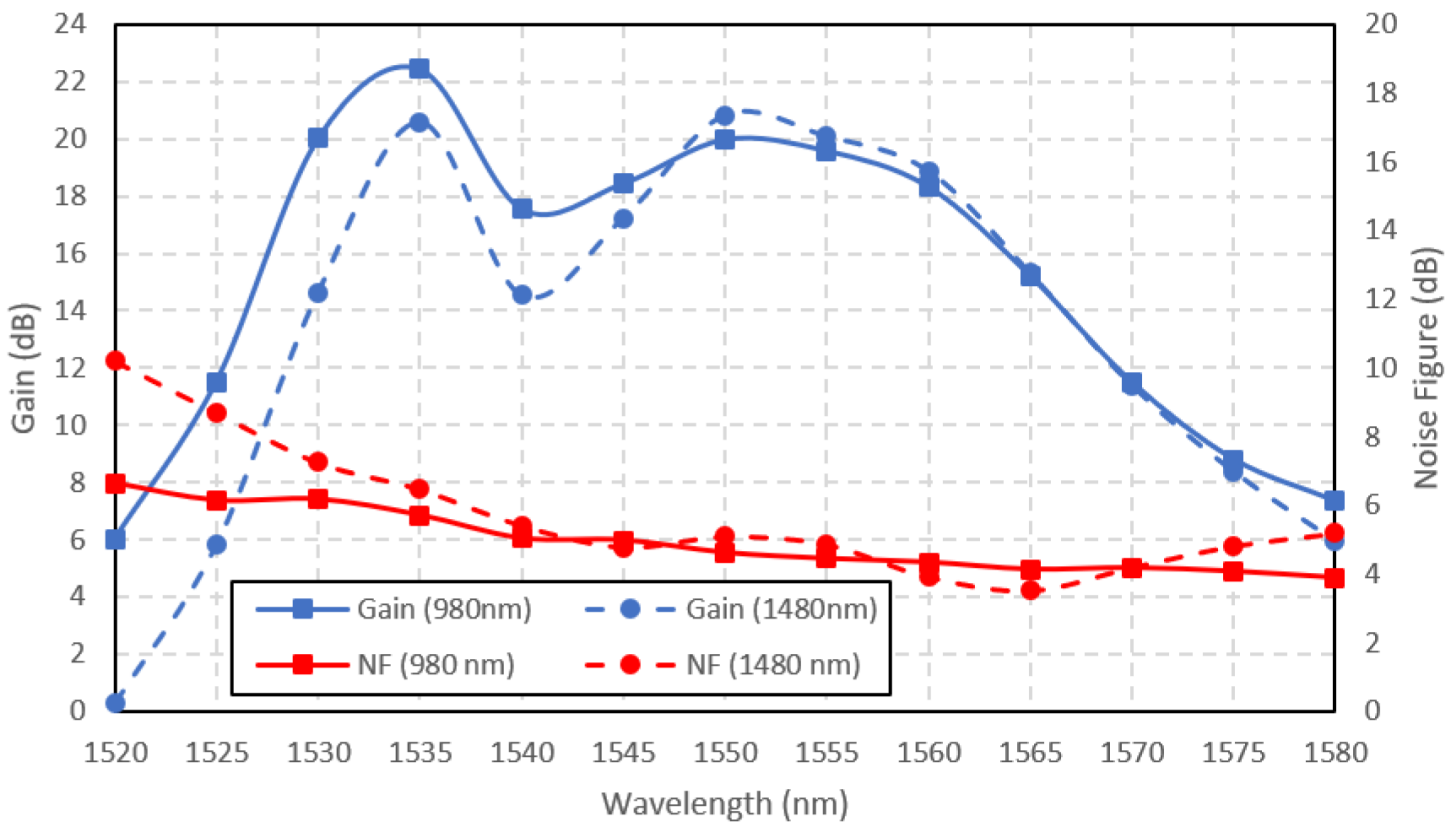
Small signal gain and noise figure at the input signal power of -30 dBm.

The comparison of the 980 nm and 1480 nm-pumped Ga-EDF amplifier small-signal gains and the noise figures are shown in Figure 6. The input signal power was set to -30 dBm. The pump power was maintained at 150 mW for both pumping wavelengths. Overall, the 980-nm pumped amplifier achieved better small-signal gain than the 1480-nm pumped amplifier, with the maximum gain of 22 dB at 1535 nm input signal wavelength. At the shorter wavelength range (1520 – 1547 nm), the 980 nm-pumped amplifier outperformed the 1480 nm-pumped amplifier, with the highest gain difference of 6 dB. At the longer wavelength range (1548 – 1580 nm), no significant difference in gain between the two pumping schemes was seen, except that the gain for 1480 nm pumping was slightly higher at the wavelength range of 1547 nm to 1565 nm, with the highest gain difference of only 0.85 dB at 1550nm. The input signal wavelength for the peak gain was related to the ASE spectra in Figure 5. The maximum gain occurs at the same input signal wavelength in the ASE spectra because it directly correlates with the ASE power. When the amplifier was forward pumped at the 980 nm wavelength, the peak gain occurred at 1535 nm, similar to the ASE peak spectra wavelength, as depicted in Figure 5. Similarly, when pumped at the 1480 nm wavelength, the peak gain occurred at 1550 nm, following its ASE spectra. The noise figure shown in Figure 6 shows that the 980-nm pumped amplifier exhibited better performance than the 1480-nm pumped amplifier at the shorter wavelength range between 1520 nm to 1545 nm. The higher noise level at the shorter wavelength is typical in EDFA due to the signal's reabsorption by the Erbium ions in the ^4^I _15/2 _state manifold. The reabsorption rate might be higher for the 1480-nm pumped amplifier due to a similar ^4^I _13/2_ metastable state lifetime.

A comparison with a previous work ( Hajireza *et al.*, 2010) that utilized EDF with nearly similar Erbium concentration and fiber length has shown that the Ga co-dopant has caused the shift of the peak gain wavelength from 1560 nm to 1535 nm. Besides that, the gain of Ga-EDF amplifier is more consistent over a wavelength range of 1530 nm to 1560 nm with a variation of only 4 dB. Whereas, the EDFA gain variation is 12 dB for the same wavelength range.

**Figure 7.**
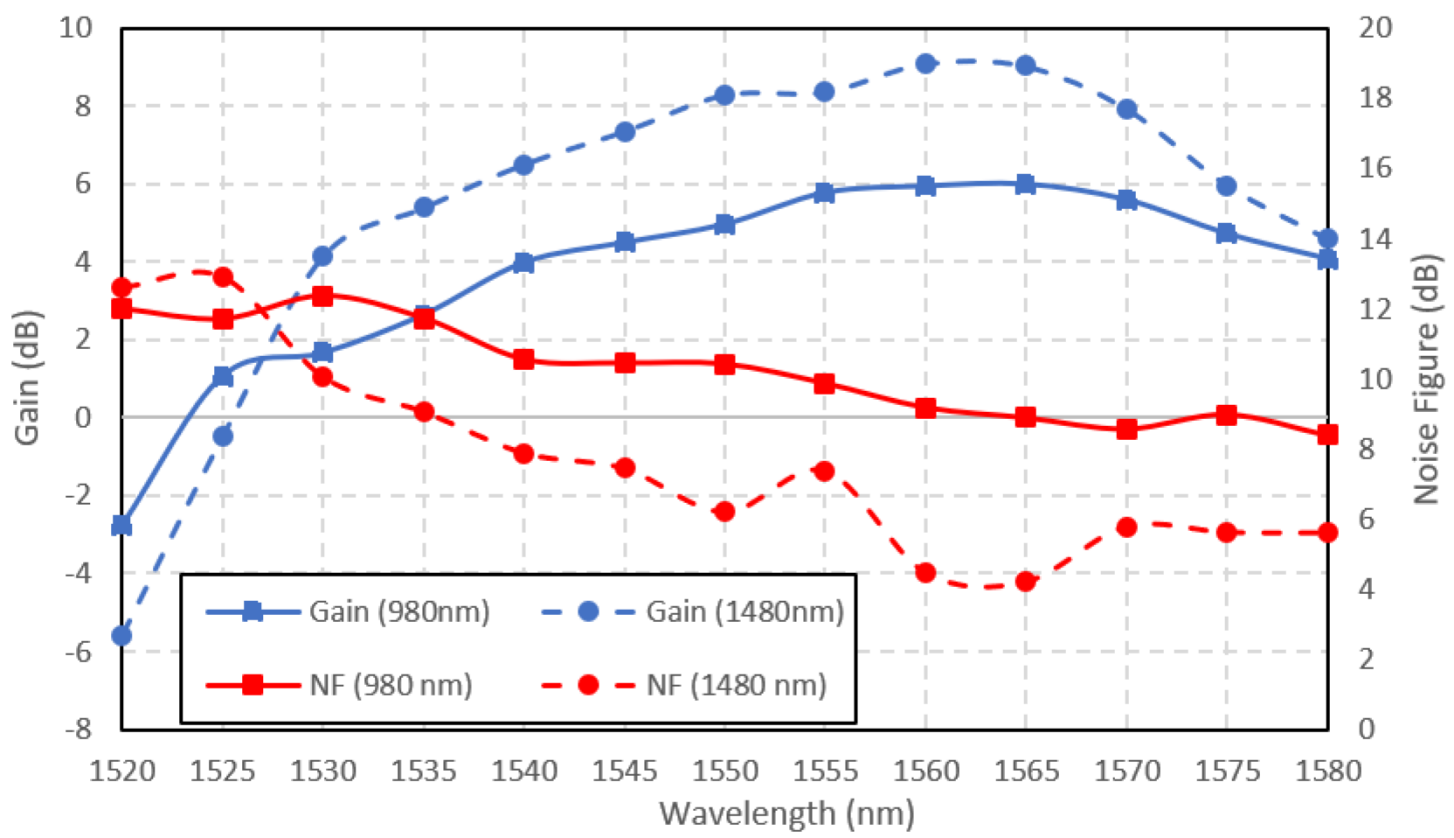
Large signal gain and noise figure at the input signal power of 3 dBm.

The Ga-EDF amplifiers' performance in amplifying large signal was investigated by setting the input power to 3 dBm. The pump power of both pumping wavelengths was maintained at 150 mW. The Ga-EDF gain and noise figure when the input signal power was set to 3 dBm is shown in Figure 7. The gain at large input signal is lower than the small-signal due to gain saturation. Contrary to the small-signal characteristics, the amplifier performed better when pumped at 1480 nm for large input signals at a longer wavelength between 1528 nm to 1580 nm. The possible reason that 1480 nm-pumped outperform the 980 nm-pumped amplifiers at large input signal was the longer fluorescence lifetime. The lifetime for the photons at metastable state when pumped at 1480 nm was 0.66% longer than the photons pumped at 980 nm, as reported by ( Dissanayake, 2015). Since pumping at 1480 nm is a direct pumping with a longer lifetime, more ions are accumulated at metastable states and contribute to the stimulated emissions. The 1480 nm-pumped amplifier outperformed the 980 nm-pumped both in terms of gain and noise figure. The highest achievable gain by the 1480 nm-pumped amplifier was 9 dB at the input signal wavelength of 1560 nm, with a corresponding noise figure of 4.5 dB. The 980 nm-pumped amplifier achieved a maximum gain of 6 dB with a noise figure of 9 dB at 1565 nm input signal wavelength.

**Figure 8.**
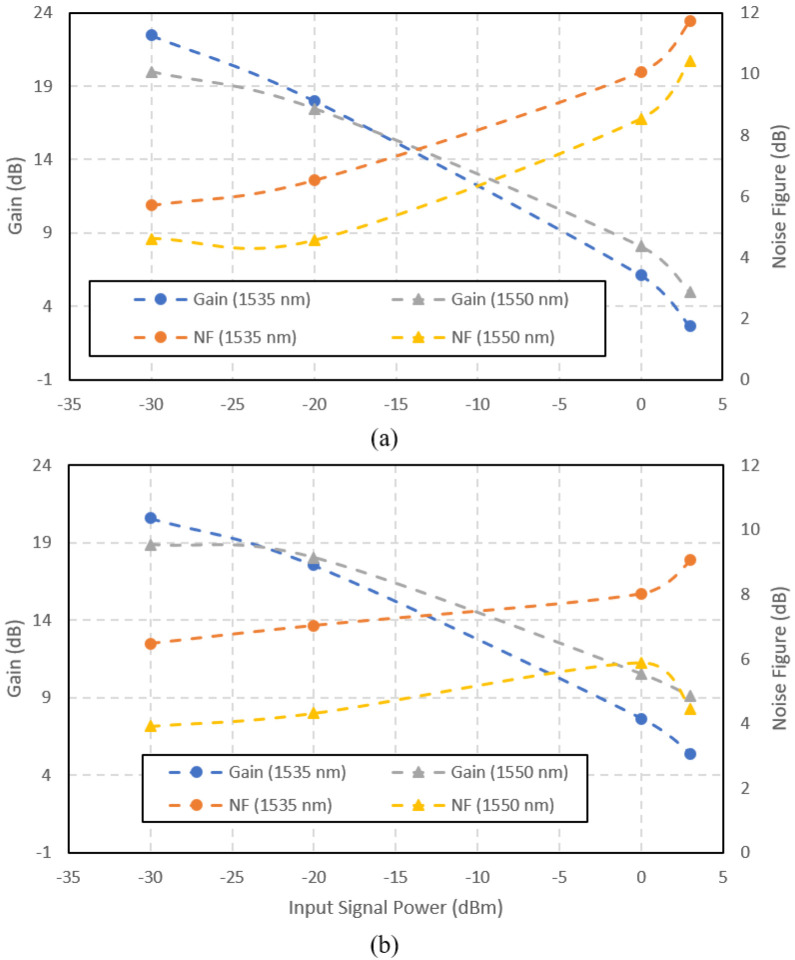
Gain and noise figure versus input power at a pump wavelength (a) 980 nm and (b) 1480 nm.

Figure 8 shows the gain and noise figure versus the input signal power for the amplifiers at the input signal wavelength of 1535 nm and 1550 nm. At high input power, the amplifier gave better performance with 1480 nm pumping than 980 nm pumping. It shows that the gain saturation point at 1480 nm pumping was higher than at 980 nm pumping. At the input signal wavelength of 1535 nm, the gain reduced drastically as the input power increased. Both pumpings result in the same trend. At the input signal wavelength of 1550 nm, the gain reduction was not as drastic as 1535 nm. It shows that the photons emission at 1535 nm was higher than 1550 nm only in the small-signal region. Above the input signal power of -20 dBm, operating at the 1550 nm signal performed better in both gain and noise figure than 1535 nm, at both pump wavelengths. The noise figure for the 980 nm-pumped amplifier was higher than the 1480 nm-pumped at all input signal power, except at -30 dBm input signal power at 1535 nm. The noise figure correlated to the ASE spectra is shown in Figure 5. The higher ASE in the 980 nm-pumped amplifier contributed to the higher noise. An obvious disadvantage of operating at 1535 nm is the high noise figure at both pump wavelengths. Even though the small-signal gain was maximum at 1535 nm input signal, the performance degraded severely as the input signal power increased. Therefore, it can be concluded that the Ga-EDF amplifier is best to be operated at the input signal of 1550 nm.

## Conclusion and further research

In this study, the gain and noise figure characteristics of the Ga-EDF amplifier pumping at 1480 nm and 980 nm wavelengths were compared. The amplifier was set up in a single pass configuration with the forward pumping scheme. The maximum gain of 22.45 dB was achieved at 1535 nm by the 980 nm-pumped Ga-EDF. However, looking at the overall performance, the 980 nm-pumped Ga-EDF only outperformed the 1480 nm-pumped Ga-EDF at the small-signal input at the wavelength range 1520 nm to 1547 nm. Both pumping's small-signal gains were comparable at the input signal wavelength range between 1547 nm to 1580 nm. Above -20 dBm input signal power, the 1480 nm-pumped Ga-EDF achieved a higher gain and lower noise figure than the 980 nm-pumped. It indicates that the gain saturation point at 1480 nm pumping is higher than at 980 nm pumping in the Ga-EDF amplifier. The 980 nm-pumped amplifier was severely affected by the ASE noise, especially at the high input signal power. Considering the overall performance, operating the amplifier at 1550 nm is better than at 1535 nm, except in the case of input power below -20 dBm. The Ga-EDF amplifier's investigation using a double-pass configuration can be considered to achieve a higher gain in the future. The use of Ga-EDF as a gain medium in a high-power laser also could be investigated.

## Data availability

### Underlying data

Figshare: ASE, gain and noise figure of Gallium-Erbum doped fiber amplifier at the C- band. https://doi.org/10.6084/m9.figshare.14213582.v1 ( Ibrahim *et al.*, 2021).

This project contains the following underlying data:

- ASE Spectra of the Ga-EDF pumped at 980 nm and 1480 nm.csv
- Gain and noise figure versus input signal power obtained at a pump wavelength 980 nm.csv
- Gain and noise figure versus input signal power obtained at a pump wavelength 1480 nm.csv
- Large signal gain and noise figure of the Ga-EDF amplifier at the input signal power of 3 dBm.csv
- Small signal gain and noise figure of the Ga-EDF amplifier at the input signal power of -30 dBm.csv

Data are available under the terms of the Creative Commons Zero "No rights reserved" data waiver (CC0 1.0 Public domain dedication).
