# Supplementary material for: Comparison of 1480 nm and 980 nm-pumped Gallium-Erbium fiber amplifier
Source: F1000Res. 2021 Sep 8;10:251. Originally published 2021 Mar 29. [Version 2] doi: 10.12688/f1000research.50952.2 (PMC11116936; doi:10.12688/f1000research.50952.2)
Supplement: Peer review history

# Reviewer report: Reviewer response for version 2

**DOI:** 10.5256/f1000research.77204.r267408

**Published:** 2024-05-23

**Authors:** Weichao Wang

**Affiliations:** 1 South China University of Technology, Guangzhou, Guangdong, China

In this study, the gain and noise figure characteristics of the Ga-EDF amplifier pumping at 1480 nm and 980 nm wavelengths were compared. The amplifier was set up in a single-pass configuration with the forward pumping scheme. The following issues are worth considering:

 1. In Figure 5, the authors point out that the ASE at 980 nm is stronger due to higher absorption in this band. However, in the author's article titled "Performance of Gallium-Erbium Fiber Amplifier in a Double-Pass Configuration" Samsudin S I, Ibrahim S A, Mansoor A, et al. International Journal of Integrated Engineering, 2024, 16(3): 92-99 | 10.30880/ijie.2024.16.03.009, the results are opposite, and the authors attribute it to the effect of reabsorption. There are many factors affecting ASE, including absorption coefficient, differences in emission processes between these two pumping schemes, reabsorption, coupling efficiency, etc. Therefore, a comprehensive consideration is needed. Additionally, Figure 5 in this paper shows only slightly higher ASE intensity at 980 nm pumping, which may not imply any significant conclusion.

 2. As pointed out by previous reviewers, the data in Figure 6 lack error bars, but the authors' response did not address this issue. Figures 7 and 8 also lack error bars, and it is recommended to add them.

 3. What are the fundamental implications of gain and noise differences at different pumping wavelengths after gallium co-doping? This aspect requires in-depth analysis in the article. Currently, the article mainly presents experimental results, and theoretical analysis needs to be strengthened.

 4. In other erbium-doped fibers, there have been many reports on the effects of these two pumping wavelengths on fiber amplifiers or fiber lasers. For example, Professor Digonnet MJF discussed this issue in his book "Rare-Earth-Doped Fiber Lasers and Amplifiers" (Stanford University. New York: Marcel Dekker. 2001). In this paper, the authors summarize only the experimental results of Ga-EDF. Can the authors combine existing literature to summarize a general rule, i.e., under what circumstances 980 nm (or 1480 nm) pumping can achieve higher gain?

 5. Some details need to be revised, such as subscripts, spelling, etc., e.g. “Gallium oxide (Ga _2_O _3_)”, and “Erbium ions Er ^3+^’s”.

 I hope the above comments will help promote the scientific significance of this paper and inspire new ideas.

Is the work clearly and accurately presented and does it cite the current literature?

Yes

If applicable, is the statistical analysis and its interpretation appropriate?

Partly

Are all the source data underlying the results available to ensure full reproducibility?

Partly

Is the study design appropriate and is the work technically sound?

Yes

Are the conclusions drawn adequately supported by the results?

Partly

Are sufficient details of methods and analysis provided to allow replication by others?

Partly

Reviewer Expertise:

laser glass, rare-earth doped glass, luminescence, fiber laser, fiber amplifier

I confirm that I have read this submission and believe that I have an appropriate level of expertise to confirm that it is of an acceptable scientific standard, however I have significant reservations, as outlined above.

# Reviewer report: Reviewer response for version 2

**DOI:** 10.5256/f1000research.77204.r93726

**Published:** 2021-09-16

**Authors:** Colin C. Baker

**Affiliations:** 1 Optical Sciences Division, Naval Research Laboratory, Washington, DC, USA

In my opinion, the paper is now suitable for indexing.

Is the work clearly and accurately presented and does it cite the current literature?

Partly

If applicable, is the statistical analysis and its interpretation appropriate?

Not applicable

Are all the source data underlying the results available to ensure full reproducibility?

No

Is the study design appropriate and is the work technically sound?

Partly

Are the conclusions drawn adequately supported by the results?

Partly

Are sufficient details of methods and analysis provided to allow replication by others?

No

Reviewer Expertise:

NA

I confirm that I have read this submission and believe that I have an appropriate level of expertise to confirm that it is of an acceptable scientific standard.

# Reviewer report: Reviewer response for version 1

**DOI:** 10.5256/f1000research.54047.r82439

**Published:** 2021-05-05

**Authors:** Abdollah Malakzadeh Fard Khangheshlaghi

**Affiliations:** 1 School of Astronomy, Institute for Research in Fundamental Sciences, Tehran, Iran

I reviewed the article “Comparison of 1480 nm and 980 nm-pumped Gallium-Erbium fiber amplifier”.  These are my questions for the authors:

 However the comparison between the two pump lasers in the medium is a necessity and attractive,

1. The comparison is for a fixed length. Does this have a physical reason?
2. The power is limited to 150mW. Why did the authors not use higher power lasers?
3. The setup benefits a single pass one directional pumping? Why forward directional pumping? Why not backward or bidirectional pumping?
4. In figure 6, what is the error bar in the graphs? It seems there is almost no difference in noise figure or small signal gain values of pumping with both wavelengths in large ranges of the spectra. Is such insensitivity for the gain length or the co-dopant (Ga) magnitude?

Is the work clearly and accurately presented and does it cite the current literature?

Yes

If applicable, is the statistical analysis and its interpretation appropriate?

Partly

Are all the source data underlying the results available to ensure full reproducibility?

Yes

Is the study design appropriate and is the work technically sound?

Partly

Are the conclusions drawn adequately supported by the results?

Partly

Are sufficient details of methods and analysis provided to allow replication by others?

Yes

Reviewer Expertise:

fiber optics, fiber sensor, FBG, distributed fiber sensors,THz generation, Ultrashort pulse science

I confirm that I have read this submission and believe that I have an appropriate level of expertise to confirm that it is of an acceptable scientific standard, however I have significant reservations, as outlined above.

# Response

**Published:** 2021-08-29

**Authors:** Siti Azlida Ibrahim

**Affiliations:** Multimedia University, Malaysia

1. The comparison is made for the optimum length at a maximum gain. The optimum length was found using the cut-back method. In the reference, Marzuki *et al.*, 2020, the amplifier's performance with different lengths has been discussed.
2. 150 mW is the maximum pump power available at the lab.
3. The forward directional configuration was chosen as it is the simplest configuration of a fiber amplifier which we felt is enough to show the applicability of the fiber.
4. Since the experiment was conducted at a relatively low pump power, the difference is not significant due to limited gain length. As mentioned in the reference Marzuki *et al.*, 2020, a longer gain medium does not produce a higher gain output at 150mW pump power.

# Reviewer report: Reviewer response for version 1

**DOI:** 10.5256/f1000research.54047.r82436

**Published:** 2021-04-06

**Authors:** Colin C. Baker

**Affiliations:** 1 Optical Sciences Division, Naval Research Laboratory, Washington, DC, USA

It was my understanding that this is a paper was meant to explore the benefit of Ga co-doping of EDF. However, there is no comparison with an erbium doped fiber without gallium co-doping. It seems that results and analysis should be done with a comparison of an erbium doped fiber of nearly identical concentration without gallium in order to ascertain its benefits or at least a mention of the differences with the co-dopant. It is also stated that Ga2O3 is a better co-dopant than aluminum but there is no information to back that up. This paper is then essentially concerned with a comparison of pumping at 980 nm vs. 1480 nm, which has been studied extensively by many authors.

 Very little information is given about the fiber itself. I had to go to the previous reference to learn more about the properties of the fiber, such as the ratio of Ga to Er (very important), and its NA, etc... There I learned that the fiber also contains phosphorous. What is its role here?

 Please check for errors. In the abstract its says signal powers from -30dBm to 3dB, it should say ...to 3dBm.

Is the work clearly and accurately presented and does it cite the current literature?

Partly

If applicable, is the statistical analysis and its interpretation appropriate?

Not applicable

Are all the source data underlying the results available to ensure full reproducibility?

No

Is the study design appropriate and is the work technically sound?

Partly

Are the conclusions drawn adequately supported by the results?

Partly

Are sufficient details of methods and analysis provided to allow replication by others?

No

Reviewer Expertise:

Synthesis and characterization of rare earth doped silica fibers

I confirm that I have read this submission and believe that I have an appropriate level of expertise to confirm that it is of an acceptable scientific standard, however I have significant reservations, as outlined above.

# Response

**Published:** 2021-08-29

**Authors:** Siti Azlida Ibrahim

**Affiliations:** Multimedia University, Malaysia

1. A comparison with an erbium-doped fiber without Gallium co-dopant is added in the discussion section (paragraph 3). The comparison was made with an erbium-doped fiber with nearly identical erbium concentration and fiber length. 
2. This work is not intended to compare aluminum co-dopant and Gallium co-dopant. The aluminum as a co-dopant is mentioned as a justification to use Gallium. The word “more” in the last sentence of paragraph 1 was removed to avoid confusion.
3. The fiber properties were added in the second paragraph of the Introduction part, including Ga:Er ratio, the NA, and the cut-off wavelength.
4. During the fabrication, phosphorous is added to increase the pore size of the deposited silica soot, hence increase the incorporation of dopants.  It will also reduce the required sintering and collapse temperatures.  For this preform case, it does not have any effect on the optical properties of the fiber.
5. Correction made in the abstract (dB changed to dBm). 
